# Notes and News

**Published:** 1902-01-04

**Authors:** 


### NOTES AND NEWS.

THERE is a certain amount of plague in Mauritius, nearly forty cases being reported last week.

PRINCESS CHRISTIAN has promised to lay the foundation stone of the country branch of the Mount Vernon Hospital for Consumption, at Northwood, in May.

THE Russian surgeon Professor Minin is using the violet rays of electric light as a substitute to cocaine in surface operations. They are reported to stimulate the healing process and to be antiseptic.

A FIRE recently occurred at the Cumberland Infirmary. A beam in a flue had smouldered for a couple of days, and finally was fanned into flame by a ventilator. The fire brigade put out the fire without difficulty, and the patients were quite unaware of the danger.

PROFESSOR HEINZ, of Erlangen, recommends those who suffer from sea-sickness to draw long and vigorous breaths at frequent intervals. This, he insists, is a complete antidote. But the very simplicity of the preventive is quite enough to keep it from being put unfailingly into practice. To continue to draw long breaths at frequent intervals for, say, 12 hours would be no mean physical feat, and we fear that the antidote will soon be discredited because few could consistently follow the directions.

THE action of the educational authorities in Chicago in requiring school teachers to pass an examination in health is much to be commended. There is more reasons than one why it is advisable that those who have the training of the young should be of good physique. In the upper class boys' schools in England the importance attached to the athletic capabilities of the masters is not without its drawbacks, and it has already produced an exaggerated veneration of athletic attainments as opposed to mental ones amongst the boys of the present generation. But undoubtedly a just balance, which would foster an understanding of the true value of both mental and physical culture, is much wanted.

THREE hundred thousand francs have been left by Madame Mangini, of Lyons, to found a sanatorium for anæmic and chlorotic women of that city.

WE regret to find the name of Civil Surgeon J. Kelso Reid amongst the list of deaths on December 26th resulting from the Tweefontein engagement.

A NEW method of killing rats by the use of carbonic acid in a liquid state has been carried out at Marseilles, where the experiments have proved highly satisfactory.

THE Archbishop of Canterbury has kindly consented to preside at the annual meeting of the After Care Association for Poor Persons discharged recovered from Asylums for the Insane, to be held at Lambeth Palace on Saturday, February 8th, 1902, at 3 P.M.

THE new Act which has now come into operation with the view of restricting the sale of intoxicants to children is being met with very varied receptions, but a large number of public houses have already placed the notice in conformity with the Act in their windows.

THE use of hypnotism as a moral regenerator is to be tested at a Children's Home in Indiana. The Home is practically a reformatory, and a hypnotist from Chicago has been engaged with the view of utilising his powers to reform these children by "suggestion."

IT is not very pleasant to realise that at any moment dwellers in cities using electricity may alight upon an escaping electric current and meet their death without warning. Yet such is the case, as shown by recent experience when a horse being driven in Hampstead was killed by an escaping current.

THE committee of the Paddington Green Children's Hospital have received from Mrs. Frank L. Wright and Miss Scott a contribution of £330 towards the fund now being raised for the new convalescent home at "Fair View," Slough, the amount being the result of the concert given by them on the 26th ulto. at the Kensington Town Hall.

THE craze for something new has suggested a tabloid restaurant to the imagination of the public. With the great ignorance which prevails in the simplest matters of hygiene it is likely that some people, to whom conveniently swallowed tabloids may seem more attractive than the protracted consumption of a meal, may be quite unaware that the presence of bulk in food is an essential to digestion.

PROFESSOR BRUNER, of New York, predicts a great change in the human race of the future. By way of omissions he mentions the absence of the vermiform appendix, some of the ribs and the little toes. He expects the stature and brain to be much enlarged and memory improved. Life he predicts will be longer. But after all none will be alive to verify or refute the forecast, so we need not trouble ourselves in the matter.

